# Exploratory Investigation of *Bacteroides fragilis* Transcriptional Response during *In vitro* Exposure to Subinhibitory Concentration of Metronidazole

**DOI:** 10.3389/fmicb.2016.01465

**Published:** 2016-09-20

**Authors:** Michele C. R. de Freitas, Juliana A. Resende, Alessandra B. Ferreira-Machado, Guadalupe D. R. Q. Saji, Ana T. R. de Vasconcelos, Vânia L. da Silva, Marisa F. Nicolás, Cláudio G. Diniz

**Affiliations:** ^1^Departamento de Parasitologia, Microbiologia e Imunologia, Universidade Federal de Juiz de ForaJuiz de Fora, Brazil; ^2^Laboratório de Bioinformática and Laboratório Nacional de Computação CientíficaPetrópolis, Brazil

**Keywords:** *Bacteroides fragilis*, RNA-seq, metronidazole, subinhibitory concentration, K-means clustering analysis

## Abstract

*Bacteroides fragilis*, member from commensal gut microbiota, is an important pathogen associated to endogenous infections and metronidazole remains a valuable antibiotic for the treatment of these infections, although bacterial resistance is widely reported. Considering the need of a better understanding on the global mechanisms by which *B. fragilis* survive upon metronidazole exposure, we performed a RNA-seq transcriptomic approach with validation of gene expression results by qPCR. Bacteria strains were selected after *in vitro* subcultures with subinhibitory concentration (SIC) of the drug. From a wild type *B. fragilis* ATCC 43859 four derivative strains were selected: first and fourth subcultures under metronidazole exposure and first and fourth subcultures after drug removal. According to global gene expression analysis, 2,146 protein coding genes were identified, of which a total of 1,618 (77%) were assigned to a Gene Ontology term (GO), indicating that most known cellular functions were taken. Among these 2,146 protein coding genes, 377 were shared among all strains, suggesting that they are critical for *B. fragilis* survival. In order to identify distinct expression patterns, we also performed a K-means clustering analysis set to 15 groups. This analysis allowed us to detect the major activated or repressed genes encoding for enzymes which act in several metabolic pathways involved in metronidazole response such as drug activation, defense mechanisms against superoxide ions, high expression level of multidrug eﬄux pumps, and DNA repair. The strains collected after metronidazole removal were functionally more similar to those cultured under drug pressure, reinforcing that drug-exposure lead to drastic persistent changes in the *B. fragilis* gene expression patterns. These results may help to elucidate *B. fragilis* response during metronidazole exposure, mainly at SIC, contributing with information about bacterial survival strategies under stress conditions in their environment.

## Introduction

The Gram negative anaerobic rod *Bacteroides fragilis*, a member of the gut microbiota, remains as one of the most important putative pathogen associated with endogenous infections (Wexler, 2007; Brook, 2010). Since 1950, antimicrobial agents have been widely used to treat different infectious diseases, leading to dissemination of antimicrobial resistance. The resistance phenomenon has dramatically increased among human pathogens, especially resident microbiota, resulting in only a few therapeutic options to treat infections related to resistant bacteria (Andersson and Hughes, 2010).

As a common clinical practice, to treat infections caused by *B. fragilis*, β-lactam drugs are usually prescribed, such as piperacillin-tazobactam or carbapenems. Besides that, metronidazole, clindamycin and even fluoroquinolones, are also frequently prescribed (Brook, 2016). In any way, metronidazole remains as drug of choice to treat anaerobic infections in which *B*. *fragilis* is involved (Löfmark et al., 2010).

Metronidazole, a nitroimidazole drug, is a non-toxic prodrug that requires the reduction of the nitro group to be converted into toxic nitro anion radical or hydroxyl amine forms. It is accepted that metronidazole enters the cell through diffusion, and in the anaerobic environment a chemical reduction occurs and the release of final products lead to a transmembrane concentration gradient (Edwards, 1993a; Sisson et al., 2000). After metronidazole intracellular reduction, inhibition of DNA replication is observed followed by DNA synthesis and RNA metabolism stop. Cell lysis may occur, suggesting that nitroradicals interact with other cell components resulting in membrane disruption (Diniz et al., 2000).

Metronidazole resistance mechanisms in *B. fragilis* are complex, but it is highly suggested the lack of drug chemical reduction due to decreased activity of cellular oxidoreductases enzymes (ferredoxins) involved in electron transference (Diniz et al., 2004). Additional mechanisms are thought to be associated with drug inactivation by aminothiol radical scavengers and radioprotectors, overexpression of *rec*A gene encoding for major DNA repair enzymes, mutation in *feo*AB gene encoding for iron transport protein, and metronidazole reduction to non-toxic amine derivative as result of *nim* genes expression (Narikawa et al., 1991; Edwards, 1993b; Carlier et al., 1997; Steffens et al., 2010; Vael et al., 2011; Veeranagouda et al., 2014).

Besides the biological drug-resistance mechanisms, the widespread sometimes inappropriate uses are to be considered. Add to that, the resident microbiota exposure to subinhibitory concentrations (SIC), which may interfere with microbial ecosystem, may also lead to changes in bacteria-bacteria and bacteria-host relationships related to pleiotropic regulation in bacterial gene expression, as an adaptive response (Bohnen, 1998; Diniz et al., 2004). Under SIC of antimicrobial drugs, *in vitro* studies reported cellular alterations related to the anaerobic bacteria morphology, physiology, and protein expression (Diniz et al., 2003; de Souza Filho et al., 2012; Freitas et al., 2015).

Considering this adaptive response to SIC of antimicrobial drugs, elucidation of bacterial genome expression would lead to a better understanding of microbial mechanisms to overcome antimicrobial chemotherapy. In this study we carried out an *in vitro* experimental design in order to evaluate transcriptional response of *B. fragilis* exposed to SIC of metronidazole, which allowed the observation differential expression of several bacterial genes related to various metabolic pathways involved in metronidazole response by anaerobes.

## Materials and Methods

### Bacteria and Routine Culture Conditions

**FIGURE 1:**
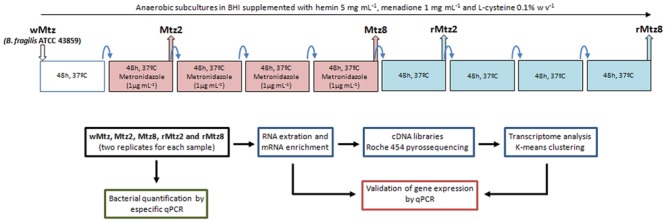
**Experimental design.** Rationale for *in vitro* bacterial growth, sampling collection points, RNA-seq approach and analyzes.

For routine culture, bacteria were grown in Brain Heart Infusion broth (Himedia, Mumbai, India) supplemented with hemin (Inlab, São Paulo, Brazil, 5 mg/mL), menadione (Inlab, São Paulo, Brazil, 1 mg/mL), L-cysteine 0.1% w/v (Inlab, São Paulo, Brazil) and metronidazole (Sigma-Aldrich, St. Louis, MO, USA), 1 μg/mL (whenever necessary), in anaerobic atmosphere (90% N_2_, 10% CO_2_), at 37°C. Cultures were let to grow until mid-log phase. As described in **Figure 1**, for *in vitro* bacterial selection, subcultures with or without metronidazole were performed in 48 h time point intervals up to 8 days. In this regard, from the parent *B. fragilis* ATCC 43859 (wMtz), the Mtz2 strain was selected after first subculture under metronidazole pressure, and Mtz8 was selected after the fourth successive subculture in the same condition. Further, rMtz2 strain was selected from Mtz8 (first subculture) after drug removal, and rMtz8 strain was subsequently selected after the fourth successive subculture in the same condition. For each subculture, a 1% inoculum was used to avoid accumulation of eventual dead cells.

### Determination of Subinhibitory Concentration (SIC) of Metronidazole

Metronidazole susceptibility pattern was determined by the broth dilution method according to the Clinical and Laboratory Standards Institute guidelines (CLSI, 2012). Briefly, 0.1 mL of a 1.0 McFarland suspension of parent *B. fragilis* strain was inoculated in Brucella broth (Himedia) tubes with increasing concentration of metronidazole ranging from 0.06 to 64 μg/mL. After 48 h of incubation, the minimum inhibitory concentration (MIC) was determined. The SIC of metronidazole was considered 0.5 MIC.

### Quantification of Bacteria

Bacterial quantification was performed by real-time quantitative PCR (qPCR). For the PCR reaction, *B. fragilis* specific primers targeting 16S rRNA gene were used: Bf (CTGAACCAGCCAAGTAGCG) and Bf-F5 (CCGCAAACTTTCACAACTGACTT) (IDT Integrated DNA Technologies). The quantitative analysis were performed on the Corbett Rotor Gene 6000 (Corbett Life Science, Mortlake, NSW, Australia) using SYBR Green PCR Master Mix (Applied Biosystems, Foster City, CA, USA) to monitor the synthesis of double stranded DNA. Reactions were assembled in a final volume of 25 μL according manufacturer’s instructions, and amplification conditions as described before (Liu et al., 2003). Each run included a standard curve in duplicate, and each sample was run in duplicate. The specificity of amplification was determined by analyzing the dissociation curves.

### RNA Extraction and mRNA Enrichment and cDNA Libraries

The total RNA was extracted from 1.0 mL of each *B. fragilis* culture by using the RNeasy mini kit (Qiagen, Venlo, The Netherlands) according to the manufacturer protocols. The RNA extracts were DNase treated (RQ1 Rnase-free Dnase Kit, Promega). The MICROBExpress^TM^ Bacterial mRNA Enrichment Kit (Ambion, Austin, TX, USA) was used to deplete the prokaryotic ribosomal RNA. The RNA quality was evaluated through on-chip microfluidic electrophoresis (Prokaryote Total RNA Pico kit, Bioanalyzer Instruments 2100, Agilent Technologies, Santa Clara, CA, USA). The RNA extracts with RNA integrity number (RIN) > 8 were considered to be of sequencing quality and cDNA libraries were then constructed using the cDNA Rapid Library Preparation Method (Roche Diagnostics, Roswell, GA, USA). In order to get an equimolar amount among libraries, we used the KAPA Library Quantification Kit (Kapa Biosystems, Wilmington, MA, USA).

### RNA-seq and Transcriptomic Data Analysis

Sequencing of cDNA libraries were carried out at Computational Genomics Unity Darcy Fontoura de Almeida (UGCDFA) at National Laboratory of Scientific Computation (LNCC, Petropolis, RJ, Brazil) using the platform Genome FLX sequencer and FLX Titanium chemistry (454 Life Sciences/Roche), according to the manufacturer’s protocols. For data analysis, firstly libraries reads were extracted and the SFF files (Standard Flowgram Format) were converted into FASTA and QUAL files. A filtering step was performed in order to remove the reads with low quality. Further, the filtered RNA-seq reads were mapped to *B. fragilis* NCTC 9343 (CR626927) reference genomic sequence using Bowtie 2 (Langmead and Salzberg, 2012), and the remaining ribosomal RNA sequences were subsequently removed using SAMtools (Li et al., 2009). It was also performed another filtering step in order to remove the replicates sequences by CD-HIT (Fu et al., 2012). Finally, the number of reads mapped to each gene was counted using HTSeq-count (Anders et al., 2015). The edgeR package from R/BioConductor was used to normalize the mapped count data (Anders et al., 2013). A Venn diagram was constructed to identify the number of shared and exclusive expressed genes in each experimental condition. Transcripts were classified based on Gene Ontology (GO)^1^ and Clusters of Orthologous Groups (COG) annotations^2^ with additional information retrieved from UniProtKB^3^ and literature. Further bioinformatics tools and clustering analysis was performed in R statistical software environment^4^. Clustering analysis was done with standard k-means from standardized expression values, with the Pearson correlation as distance measure (Brinsmade et al., 2014; Cornforth et al., 2014). Additionally, from clustering dataset, a gene expression analysis was performed using the Bioconductor package edeR (Robinson et al., 2010), in order to get the Log fold-change (LogFC) and *P*-value statistics for comparisons between each pair of treatments.

### DNA Sequencing Validation

To validate the transcription levels obtained from RNA-seq, real-time quantitative PCR (qPCR) was performed to compare gene expression patterns. The RNA extracts were used as template. Six representative genes of those identified by RNA-seq were tested (**Table 2**). For cDNA synthesis, ImProm-II^TM^ Reverse Transcription System (Promega) was used. The qPCR experiments were performed using Corbett Rotor Gene 6000 (Corbett Life Science) with three technical replicates for each sample. Reactions were conducted in 25 μL reaction mixtures containing 1 μL of diluted cDNA, 2X Rotor-gene SYBR Green PCR Master Mix (Qiagen, Valencia, CA, USA), and gene-specific primers (**Table 2**). The qPCR reactions included initial heating for 10 min at 95°C, followed by 40 cycles of 95°C, 10 s; 60°C, 15 s; 72°C 20 s. To calculate fold changes, the 2^-ΔΔCt^ method was used with the constitutively expressed 16S ribosomal RNA gene as a control for normalization.

### Data availability

A NCBI BioProject was created (accession number PRJNA338776). The expression values (fold-changes relative to each condition) are available in Additional File 3, and raw data is available from the corresponding authors upon request.

## Results and Discussion

### Overview of the *B. fragilis* Transcriptome by RNA-seq Approach

**FIGURE 2:**
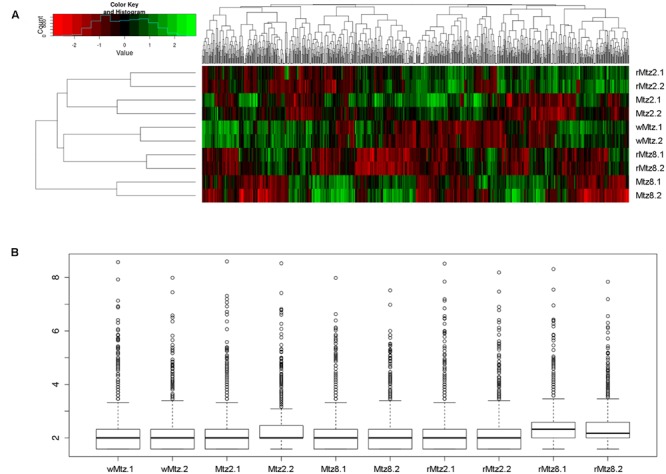
**Gene relative expression analyses. (A)** Heatmap showing the relative expression levels of each transcript (column) in each sample (row). The image was prepared in R (http://www.r-project.org). **(B)** Boxplot displaying the relative expression patterns to normalized replicates.

The *B. fragilis* strains used in this study are listed in **Table 1** and the experimental design is presented in **Figure 1**. Considering the minimal inhibitory concentration (MIC) determined as 2 μg/mL for the parent strain (wMtz), in agreement with CLSI (CLSI, 2012), SIC of metronidazole was defined as 1 μg/mL (0.5 MIC). A reduced sensitivity to metronidazole was observed for *B. fragilis* strains selected *in vitro* after successive subcultures in either presence or absence of drug (+2log_2_ MIC). Results from bacterial quantification showed that metronidazole was not able to interfere with *B. fragilis* population levels. Quantification values based on qPCR targeting to 16S rRNA gene ranged between 8.57 (± 0.22) and 8.55 (± 0.06) log copy/mL if considering the initial cultures under metronidazole pressure and the further culturing without drug, respectively. The purpose of this study was to determine genome-wide RNA profiles in *B. fragilis* either by the response to the stress imposed by SIC of metronidazole, as well as after drug removal. Sequence reads were mapped to the annotated *B. fragilis* NCTC 9343 (CR626927) genome. The pattern of relative gene expression was similar between the two biological replicates with a correlation coefficient of *r* = 0.97, *r* = 0.96, *r* = 0.75, *r* = 0.98, *r* = 0.96 for *B. fragilis* ATCC 43859 considering each tested condition, respectively (**Figure 2**).

**Table 1:** Strains used in this study and metronidazole susceptibility patterns.

Strains	Phenotype	MTZ MIC^a^ (μg/mL)	Reference
wMtz	Parent *Bacteroides fragilis* ATCC 43859 (wMtz.1 and wMtz.2 in supplementary files, after two replicates).	2.0	Myers et al., 1987
Mtz2	Strains derived from wMtz selected after first subculture in SICs of MTZ, 1 μg/mL (Mtz2.1 and Mtz2.2 in supplementary files, after two replicates).	8.0	This study
Mtz8	Strains derived from wMtz selected after fourth successive subculture in SICs of MTZ (Mtz8.1 and Mtz8.2 in supplementary files after two replicates).	8.0	This study
rMtz2	Strains derived from Mtz8 selected after first subculture in absence of MTZ (rMtz2.1 and rMtz2.2 in supplementary files after two replicates).	8.0	This study
rMtz8	Strains derived from Mtz8 selected after fourth successive subculture in the absence of MTZ (rMtz8.1 and rMtz8.2 in supplementary files, after two replicates).	8.0	This study

*^a^MTZ, metronidazole; MIC, minimal inhibitory concentration determined by broth dilution method according CLSI guidelines for anaerobic bacteria (CLSI, 2012).*

The expression analysis demonstrated that approximately 93% of the *B. fragilis* ATCC 43859 reads mapped to *B. fragilis* NCTC 9343 genome. The reads mapping to the reference genome revealed a total of 2,146 expressed genes among all samples of *B. fragilis* ATCC 43859, which represented 51% of complete protein coding genes annotated in this bacterial strain (**Supplementary Table S1**). From the expressed genes, a total of 1,618 (77%) were assigned to a Gene Ontology term (GO), biological process, molecular function or cellular location term, suggesting that most of the known cellular functions were identified. The total number of genes identified per each sample was equal to 843, 1032, 856, 939, 1052 for wMtz, Mtz2, Mtz8, rMtz2, rMtz8, respectively (**Supplementary Table S1**).

**FIGURE 3:**
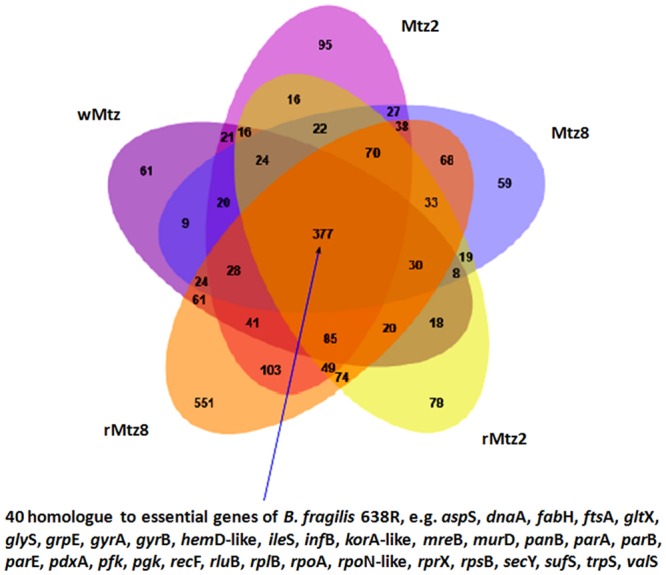
**Venn diagram.** Overview of shared or exclusive gene expression in all tested strains. It was highlighted the number of genes in the core, which were found to be homologous to the strain 638R. The image was prepared with jvenn, a JavaScript library (Bardou et al., 2014).

We also determined the number of expressed genes shared among all strains, which were 377 genes that may be regarded as a common core critical for *B. fragilis* survival. Recently, other authors determined that among the 4,326 protein coding genes of *B. fragilis* 638R, 550 were essential for its survival (Veeranagouda et al., 2014). So, we examined these 550 essential genes and compared to the shared core determined in this study (**Figure 3**; **Supplementary Table S2**). In fact, this revealed 40 genes that are homologous between both divergent strains, which include most housekeeping COG functions, such as translation, ribosomal structure, and biogenesis (12 genes), DNA replication, recombination, and repair (five genes), cell division and chromosome partitioning (three genes), transcription (three genes); coenzyme metabolism (three genes) and post-translational modification (two genes), among others (**Figure 3**).

Considering the wild type strain (wMtz), only 61 genes were exclusively expressed, whereas 154 genes (Mtz2 and Mtz8) were expressed exclusively under metronidazole pressure. After drug removal (rMtz2 and rMtz8) the number of exclusively expressed genes increased to 629. By comparing the exclusive genes from wMtz, Mtz2 and Mtz8, no shared COG or common GO function were observed. With regards to bacteria selected under drug pressure (Mtz strains) and those selected after drug removal (rMtz strains), 27 shared COG or common GO functions were observed (**Supplementary Table S2**). Besides that, by comparing exclusive genes from wMtz and rMtz strains, shared COG or common GO functions were only attributed to proteins of uncharacterized functions. These observations may suggest, at the end, that the strains collected after metronidazole removal (rMtz) might be functionally more similar to those cultured under drug pressure (Mtz). Although we tested SIC of metronidazole, previous studies suggested a complex shift in *B. fragilis* metronidazole resistant-strain resulting in both down- and up-regulation of expressed proteins (Diniz et al., 2004).

In this regard, our results show that even when the growth conditions are restored, similarly to the initial state without drug, the resulting strain, having gone through the SIC of metronidazole selective pressure, had drastic changes in their whole gene expression patterns (**Figure 2**).

### *B. fragilis* Genome-Wide RNA Profiles in Response to Subinhibitory Concentration of Metronidazole

It is discussed the efficiency regarding GS FLX 454 Roche platform when considered to transcriptomic experiments, if compared to other methodological approaches (D’Amore et al., 2016). In order to overcome, issues related to the coverage limitation, once GS FLX 454 Roche platform was the only infrastructure available, we have chosen to explore the whole normalized RNA-seq data from the five sampling points to identify genes that potentially contribute to the response to SIC of metronidazole. The normalization was determined by the k-means clustering and validated by qPCR gene expression assays since the biological replicates showed a good correlation (Brinsmade et al., 2014; Cornforth et al., 2014).

**FIGURE 4:**
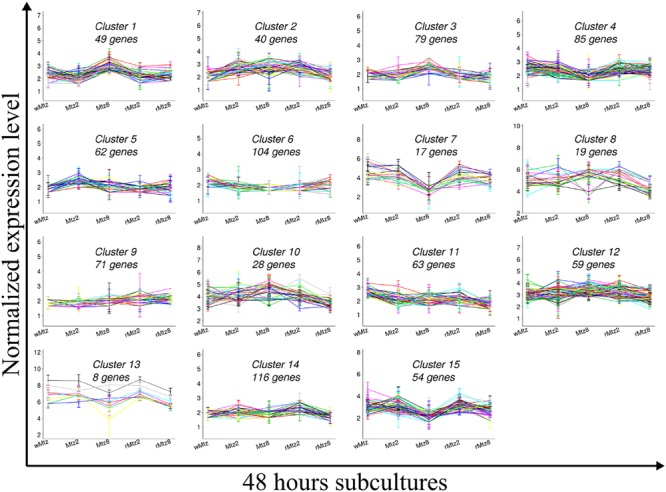
**K-means clustering.** Graded response to changes in 48 h of anaerobic subculture of *Bacteroides fragilis* ATCC43859 revealed by k-means clustering set to 15.

Thus, the dataset was subjected to k-means clustering, set to 15 to identify distinct expression patterns (**Figure 4**; **Supplementary Table S3**). To determine up and down-regulations along to their statistical significance (*P* ≤ 0.05) between treatments, a differential gene expression analysis was performed. **Figure 4** indicates the normalized expression levels of the clusterized genes for all experimental conditions over 48 h subcultures. Inside each of the 15 clusters, the number of genes per cluster is presented. In general, genes located in the same operon or with related functions fell into the same cluster or at least two clusters, supporting the number of clusters used in the calculations. For example, several genes involved in the peptidoglycan biosynthesis pathway (i.e., *mur*EDC, *mra*Y) fell into cluster 14, whereas the gene *mraY* was designated to cluster 9. Genes encoding for the ATP synthase (i.e., *atp*DGA) operon were assigned to clusters 12 and 4. Genes involved in the leucine biosynthetic process (i.e., *leu*ABC) were assigned to clusters 11 and 6, and genes encoding for the acetyl coenzyme A carboxylase (ACC) complex (i.e., *acc*CD) fell into cluster 12.

Therefore, based on the gene expression patterns revealed by K-means clustering and qPCR validation (section after) we shall discuss the main metabolic pathways which were already described in the literature as being involved with *B. fragilis* response to SIC of metronidazole.

As long as metronidazole has a nitro group from its 5-nitroimidazole structure that must be reduced for the drug to become active inside prokaryotic cell, the resulting substances may be related to strand DNA breakage, including the possible inhibition of DNA repair mechanisms (Diniz et al., 2000). Firstly, the reduction occurs in one-electron step where the nitro moiety RNO2 is reduced to form a superoxide anion (RNO^2-^), and subsequent steps form a nitrous derivative (RNO), to a nitrous free radical (RNO^-^) and a hydroxylamine derivative (RNHOH) (Veeranagouda et al., 2014). The pyruvate-ferredoxin oxidoreductase (PFOR) is a key enzyme required for the reduction and activation of MTZ (PFOR-dependent activation; Leitsch et al., 2011). The enzyme is metabolically involved in the pathway of oxidative decarboxylation of pyruvate with the participation of thiamine followed by the transfer of an acetyl moiety to coenzyme A, for the synthesis of acetyl-CoA (Kletzin and Adams, 1996). We have identified three genes encoding for the subunits alpha (BF9343_4129), beta (BF9343_4130), and gamma (BF9343_1572) of PFOR, being alpha and beta subunit-encoding genes assigned to cluster 6 whereas gamma subunit-encoding gene to cluster 12 (**Supplementary Table S3**). The fact of these genes grouped into two clusters could be explained once the genes encoding for the subunits alpha and beta are belonging to the same operon, whereas the corresponding gamma subunit-encoding gene is part of another operon altogether the ORF BF9343_1571 (Pertea et al., 2009).

The expression of PFOR genes showed enhanced levels in the subcultures collected after drug exposure (Mtz2 and Mtz8) and decreased levels in both the starting culture (wMtz) and after the drug removal (rMtz2 and rMtz8; **Supplementary Table S3**; **Supplementary Figure S1**). Down-regulation of PFOR genes have already been associated to metronidazole resistance in both *B. fragilis* and other bacteria (Diniz et al., 2004; Leitsch et al., 2011).

Among another enzymatic systems described in anaerobic microorganisms thought to be involved in metronidazole activation, the nitroreductases catalyze the reduction of nitro groups and in the metabolic process oxidize NAPH to NADP^+^ (nitroreductase-dependent activation; Sisson et al., 2000; Leitsch et al., 2011). In our experiments, a gene (BF9343_0189) encoding for an oxygen-insensitive NAD(P)H nitroreductase showed high expression level in subcultures grown under pressure of SIC of metronidazole and was related to cluster 10 (**Supplementary Table S3**).

Although, the 200 amino acid sequence of BF9343_0189 showed low similarity (BLASTp > 30%) with either NfsB of *Escherichia coli* or RdxA of *Helicobacter pylori*, it contains a nitroreductase domain (InterPro ID IPR029479, from S4 to K200) and the nitroreductase family signature (Pfam ID PF00881 from K10 to G178) which highly suggests to be a putative nitroreductase involved in the reduction of nitrogen-containing compounds, including those containing the nitro functional group. This response of high PFOR and nitroreductase expression levels were expected, since the wild-type *B. fragilis* strain used in our experiments is not metronidazole-resistant. Otherwise, metronidazole reduction inside de *B. fragilis* cells should become active.

On the other hand, a metabolic shift away to PFOR-dependent activation pathways is related to conversion of pyruvate to lactate via lactate dehydrogenase (LDH). This is another pathway that avoids the activation of nitroimidazoles by reducing pyruvate to lactate, instead of its metabolism to acetyl-CoA. Indeed, it was already observed in *B. fragilis* high LDH activity compensating for decreased pyruvate ferredoxin oxidoreductase activity related to metronidazole resistance (Narikawa et al., 1991; Diniz et al., 2004). Also, similar mechanism was observed in *H. pylori* (Kaihovaara et al., 1994). From our k-means clustering analysis, we identified in the cluster 13, the ORF BF9343_0467 (**Supplementary Table S3**) encoding for an enzyme LDH belonging to L-lactate/malate dehydrogenase family (InterPro ID IPR001557) that catalyzes the reversible NAD-dependent interconversion of pyruvate to L-lactate. As demonstrated in **Supplementary Figure S1**, the expression intensity of BF9343_0467 was higher than the expression intensity of other three genes encoding for the PFOR. Consequently, since this experiment was carried out at anaerobic condition, we may suggest that under SIC of metronidazole, *B. fragilis* (Mtz2 and Mtz8) are expressing different levels of pyruvate metabolizing enzymes.

As mentioned above, after metronidazole activation, the nitro radical-anion intermediate derivatives may become a reactive cytotoxic species and could be impaired by aminothiol radical scavengers and radioprotectors, normally present in the cell (Edwards, 1980). Regarding this type of protection against the radical-anion intermediate derivatives, we identified a few genes with high expression level during metrodidazole exposure, such as the *sod*B gene (cluster 8, **Supplementary Table S3**) encoding for a superoxide dismutase (SOD), which is a major enzyme produced by microorganisms to evade the potentially damaging reactive oxygen species (ROS). SODs are metalloenzymes that use a redox-active metal to unbalanced two molecules of superoxide (O^2-^) to oxygen (O_2_) and hydrogen peroxide (H_2_O_2_), the latter of which can be removed by catalase and peroxidase enzymes (Broxton and Culotta, 2016).

In Gram-negative bacteria, two intracellular cytosolic SODs (Mn-SodA and Fe-SodB) play important roles in the removal of intracellular superoxide anion (O^2-^) (Broxton and Culotta, 2016). Considering the intermediate superoxide anion (RNO^2-^) generated after metronidazole intracellular reduction, it has been reported an increased expression of iron-containing superoxide dismutase (Fe-SOD, *sod*B) in *Entamoeba histolytica* (Wassmann et al., 1999).

Again, once our experiments were carried out at anaerobic conditions, a putative functional Fe-SodB would be transferring the two molecules of superoxide anion (O^2-^) to hydrogen peroxide (H_2_O_2_). Besides, the gene *kat*A encoding for a catalase (cluster 15, **Supplementary Table S3**), as well as the ORF BF9343_3557 encoding for a putative peroxidase enzyme (cluster 14, **Supplementary Table S3**), both showed high expression level under drug selective pressure (Mtz2 and Mtz8). Same phenomenon was observed considering the *ahp*C gene (BF9343_1149 in cluster 2) encoding for another predicted peroxidase enzyme, in particular a peroxiredoxin (**Supplementary Table S3**; Choi et al., 1998). The *ahp*C encodes for an AhpC protein that is a potent antioxidant stress-related enzyme converting hydrogen peroxide (H_2_O_2_) to water (H_2_O). AphC protein levels have been observed to be increased during metronidazole exposure in *H. pylori* (McAtee et al., 2001). Interesting, the expression level of *ahp*C is activated by hydrogen peroxide, which would explain our results (Rocha and Smith, 1999). At that moment, our findings may suggest that under stress imposed by SIC of metronidazole, one of the *B. fragilis* responses may be to destroy superoxide radical anion (RNO^2-^) via the Fe-SOD pathway and peroxidase enzymes. Metronidazole resistance related to oxidative stress has already been reported for *H. pylori*. In this situation, the oxidative stress response is mediated through homeostatic regulator HsrA. SOD showed an inverse relationship between HsrA levels and SOD activity, suggesting that HsrA may serve as repressor of *sod*B, while activate oxidative defense genes in response to low levels of drug toxic metabolites, before becoming available to interact with cellular components (Olekhnovich et al., 2014).

On the other hand, among the bactericidal effects related to metronidazole, it is the generation of single-stranded (ss) and double-stranded (ds) DNA breaks (Edwards, 1980; Diniz et al., 2000; Sisson et al., 2000). Several studies have been performed in order to analyze the role of DNA repair proteins in the response to the metronidazole treatment (Casanueva et al., 2008; Steffens et al., 2010). It was already been shown that *B. fragilis* strain overexpressing the RecA protein exhibited increased resistance to this drug, being the RecA a major DNA repair protein that performs homologous recombination repair and controls the SOS response (Steffens et al., 2010).

The double-strand-break repair (DSBR) model begins with processing of the linear duplex DNA at the double-strand-break (DSB) to generate the ssDNA, needed for DNA strand invasion of a dsDNA homolog by RecA protein (Szostak et al., 1983). In case of RecA protein fails to assemble on the ssDNA produced, then accessory proteins RecF, RecO, and RecR can assist this assembly step (Umezu and Kolodner, 1994). From our k-means clustering analysis, *rec*A gene (BF9343_1121), assigned to cluster 11 (**Supplementary Table S3**), showed low expression level during the subculture with SIC of metronidazole, if compared to the subculture without the drug, and further, showing lowest expression level after removal of the drug (**Supplementary Figure S2**). In this analysis, we have also identified the *rec*F and *rec*N genes, which were assigned to the clusters 14 and 3, respectively (**Supplementary Table S3**). **Supplementary Figure S2** also shows the gene expression profile of *rec*F and *rec*N genes, which differs between them as well as when compared with the profile of the *rec*A gene. While the *rec*N gene showed an increased expression level in the second subculture under metronidazole exposure (Mtz8) when compared to the subculture without the drug (wMtz), the *rec*F gene had increased expression at both the first metronidazole subculture (Mtz2) and then immediately after drug removal (rMtz2).

Firstly, these results suggest that the RecA protein would not be completely involved in the DNA repair at SIC of metronidazole, but in that situation, both RecF and RecN would be functioning. However, RecF would be initially functional as long as metronidazole enters the bacterial cell, and until very shortly after the drug has been removed or degraded.

Regarding the RecN protein function, it has been demonstrated in *Deinococcus radiodurans* that it is needed in the DSBR, stimulating the intermolecular association of linear duplex DNA (Reyes et al., 2010). Meanwhile, it has been showed that expression of the RecN protein may vary widely among bacteria. For example, in *E. coli* expression of RecN is highly regulated and it is thought unlikely that has a role in the early steps of DSB repair (Meddows et al., 2005). Add to that, for *D. radiodurans*, it is suggested a housekeeping role for RecN in genome maintenance, even though it is required for DNA damage tolerance (Funayama et al., 1999; Kidane et al., 2004; Tanaka et al., 2004). According to our results we may suggest that in *B. fragilis*, the *rec*N gene seems to be required for DNA damage tolerance after treatment with SIC of metronidazole.

Other proteins involved in DNA damage repair observed in our experiments were UvrA and UvrB, which belong to the excision nucleotide repair (NER) pathway (Kuzminov, 1999). In *E. coli* it has been shown that NER system is involved in DNA damage repair associated to metronidazole action (Jackson et al., 1984). **Supplementary Figure S3** shows the expression intensity of genes *uvr*A1, *uvr*A2 and *uvr*B, which have been associated with the clusters 11, 12, and 6, respectively (**Supplementary Table S3**). It is clearly noticeable that the *uvr*A2 gene is the only one whose expression is increased upon drug exposure (Mtz2 and Mtz8).

The comparison between the amino acid sequences of UvrA1 and UvrA2 reveals low identity (BLASTp, 46%), differing in the functional domain architecture. While UvrA2 contains two domains ABC transporter-like (IPR003439; Y339 to L575 and R587 to Q919), UvrA1 carries only one (G608 to R936). According to their domain architecture, it is accepted that UvrA consists of two ATP-binding domains separated by a linker region, and in turn, each of these contain a Walker A motif (A1 and A2), a Walker B motif (B1 and B2), and an ABC-signature sequence (ABC1 and ABC2) (PDB entry: 2R6F) (Pakotiprapha et al., 2008). Then, in view of this UvrA topology, it seems that the functional copy may be the *uvr*A2 gene instead of *uvr*A1, as it showed to be overexpressed in our experiments. Functionally, UvrA is the first protein in the NER cascade, initially detecting distortions in the DNA double helix. The knowledge about the UvrA molecular function is based on the crystal structure of an UvrA dimer in complex with damaged DNA, which allows UvrA to distinguish damaged from non-damaged DNA (Jaciuk et al., 2012).

Recently, it has been reported that UvrA performs two different mechanisms in order to detect a potential DNA lesion within a large extension of non-damage DNA, which are UvrB-dependent and UvrB-independent (Kad et al., 2010). Namely, UvrA alone can perform a three-dimensional searching by only short-term binding events and jumping between different DNA molecules. Otherwise in the presence of UvrB, UvrA forms an UvrAB complex applying a one-dimensional search by sliding along the DNA (Kad et al., 2010). By the results shown here, possibly UvrA is performing a three-dimensional searching for detecting a possible DNA damage in an UvrB-independent way rather than a one-dimensional searching through an UvrAB complex. The *uvr*B gene is less expressed than *uvr*A under metronidazole pressure and in the first subculture after drug removal (**Supplementary Figure S3**).

Different authors have demonstrated in *B. fragilis* another gene playing a role in resistance against metronidazole and other DNA damaging agents, namely *reg* (BF9343_3162) encoding for an AraC-family regulatory proteins (Casanueva et al., 2008). In our experiments, the expression analysis of *reg* gene (BF9343_3162 or BF3248, assigned to cluster 2, **Supplementary Table S3**) showed a different expression profile compared to *rec* or *uvr* genes displaying increased expression intensity upon drug exposure, and still after 48 h after drug removal (**Supplementary Figure S3**). Also, by our K-means clustering, the *reg* expression profile was different from those reported for the homologous gene (BF638R3281) in *B. fragilis* 638R, in which it is expressed constitutively.

Furthermore, previous studies had demonstrated diminished expression of bacterioferritin-encoding gene (*bfr*; Diniz et al., 2004). In *H. pylori* and *E. coli*, Bfr or Dps (DNA protection during starvation protein) is known as a protein involved in DNA protection during the oxidative stress by sequestering intracellular Fe^2+^ and storing it in the form of Fe^3+^, where one hydrogen peroxide oxidizes two Fe^2+^, preventing hydroxyl radical production by the Fenton reaction (Nair and Finkel, 2004). Although, these bacteria are considered microaerophilic (*H. pylori*) or facultative anaerobic (*E. coli*), we still wanted to investigate the expression of the *bfr* or *dps* homologous gene in *B. fragilis* under anaerobic conditions. Therefore, we also check over the expression level of bacterioferritin like-encoding genes and verified the ORF BF9343_1253 (cluster 15, **Supplementary Table S3**), which has homology (BlastP, similarity > 55% and coverage > 80%) whit *H. pylori dps* gene (HP_0243) and showed lower expression intensity during SIC exposure of metronidazole if comparing to wMtz or rMtz (**Supplementary Table S3**). This observation is in agreement with literature data (Diniz et al., 2004) and may suggest that the DNA protection during the oxidative stress imposed by metronidazole pressure should not exclusively depend on bacterioferritin-like protein.

According to the literature, the efficiency of the metronidazole action on DNA might depend on the degree of strand breakage and aminothiol protection efficiency (Edwards, 1980). Hence, our results may suggest an important role of peroxidases and Fe-SOD in bacteria protection against nitro radical-anions, rather than only for DNA repair systems.

In spite of the genes described above, whose expression patterns showed a high intensity during metronidazole exposure, suggesting their involvement in resistance, others might also be involved, such as those encoding for multidrug (MDR) eﬄux pumps, e.g., BF9343_0579, BF9343_2447, BF9343_2448 and BF9343_2449, and all fell into cluster 14 (**Supplementary Table S3**). The BF9343_0579 encodes for a putative AcrB, which is a member of AcrAB multi-drug eﬄux system that is believed to protect the bacterium against hydrophobic inhibitors; BF9343_2447, BF9343_2448, and BF9343_2449 that were more expressed during metronidazole exposure (**Supplementary Table S3**) codify for the three components of a homologous *E. coli* α-hemolysin (HlyA) type I secretion system, TolC, HlyD, and HlyB, respectively (Ma et al., 1993). With regards to metronidazole, active eﬄux systems had already been associated to *H. pylori* resistance, although this is not an anaerobic bacterium, such as *B. fragilis*. The eﬄux systems were found to be expressed in multiple *H. pylori* isolates *in vitro* and are related to outer membrane eﬄux proteins (OEPs: TolC), periplasmic eﬄux proteins (PEPs: AcrA, EmrA, HlyD), and inner membrane eﬄux proteins (IEPs: AcrB) (Amsterdam et al., 2005; Liu et al., 2008). Considering TolC homologous eﬄux pumps (HP0605, HP0971, HP1327, and HP1489) (Mehrabadi et al., 2011), a high amino acid sequence similarity was observed between BF9343_2447 and HP1489 (BlastP, similarity 49%, query coverage 83% and subject coverage 84%), which adds more evidences for the results observed in this study. Indeed, the protein sequences of BF9343_2447, 9343_2448 and BF9343_2449 contain TolC family signature (from L29 to I486), HlyD domain (from A212 to G324) and ATP-binding cassette transporter signature (from C41 to A386), respectively.

Interestingly, besides to secrete α-hemolysin (HlyA), this eﬄux system can also secrete heterologous proteins. Actually, almost 400 proteins are secreted *in vitro* by *E. coli* via the hemolysin secretion pathway, even though they are entirely unrelated to HlyA (Gentschev et al., 2002). Particularly, similar to metronidazole, HlyA is a hydrophilic protein, so in that sense, we would hypothesize that in *B. fragilis* the hemolysin secretion pathway, whose genes are activated in response to metronidazole, could be mediating the drug eﬄux (Stanley et al., 1998). In opposition, other genes showed low expression intensity under SIC of metronidazole. For example, genes encoding for *N*-acetyl-beta-hexosaminidase subunits (BF9343_1729 and BF9343_1731), an oxidoreductase, were grouped into cluster 6 (**Supplementary Table S3**).

The *N*-acetyl-beta-hexosaminidase is related to binding and deglycosylation of *O*-glycosylated peptides from mammals and this function corresponds to a virulence factor, which was already been demonstrated in *Streptococcus pneumoniae* and *B. fragilis* (Rao et al., 2006; Cao et al., 2014; Shi et al., 2014). This unheard result would be considered advantageous from the clinical viewpoint, since metronidazole would suppress that virulence gene and thus it would be of interest further *in vivo* mutagenesis assays.

### Validation of RNA-Seq Data

We chose six direct targets of expressed genes to validate RNA-seq results by quantitative PCR (qPCR) expression analysis (**Table 2**). We used two sampling points, parent wMtz strain, and after 8 days SIC of metronidazole exposure (Mtz8.1 and Mtz8.2), with three technical replicates for each sample. The target genes fell into clusters 3, 6, 10, 11, and 13, respectively (**Supplementary Table S3**). Transcript abundance was normalized to that of 16S rRNA after confirmation that its expression was not significantly affected under the experimental conditions. Fold-regulation (i.e., the ratio of transcript abundance in the sampling point with metronidazole to that in the sampling point after removal of metronidazole) showed good agreement between the qRT-PCR and RNA-seq experiments (**Table 3**).

**Table 2:** Primer sequences used to validate the gene transcription levels obtained by RNA-seq approach.

Locus ID	Gene/ORF name	Sequence primers (5′–3′)
BF9343_0382	gadB	F-TGGCAGATTGAGATGCGTCA
		R-CAGACCGGTCCATGTAACCC
BF9343_3128	BF9343_3128	F-CTCATCCATCCTGACCGACG
		R-GCAGTTCGGAGTTTTGCTGG
BF9343_2131	pyrD2	F-CAGGGTTGGCAGTTTTTCG
		R-ACCCGGTACAGACGAGTACA
BF9343_3319	gdhB2	F-TCTCCACGCGGAAAGTCAAA
		R-GTCGGTTTCAGGACCGATGT
BF9343_3934	BF9343_3934	F-TGTCACTGAATTGAGGGCGT
		R-ACCATCCGAAAGACAGACCG
BF9343_0081	BF9343_0081	F-CACATGTCCATAGCCGTCGT
		R-CTGATCGCCCTTCATCCACA

**Table 3:** Validation of RNA-seq data by qRT-PCR.

Target ID	Product description	Expression by qRT-PCR,^∗^ %	Expression by RNA-seq,^∗^ %	Response to Mtz exposure
BF9343_0382	Glutamate decarboxylase beta	41.03	81.45	Downregulation
BF9343_3128	RNA polymerase sigma-E factor	42.34	88.42	Downregulation
BF9343_2131	Dihydroorotate dehydrogenase	39.69	74.94	Downregulation
BF9343_3319	Glutamate dehydrogenase	26.88	86.13	Downregulation
BF9343_3934	Related surface antigen protein	464.12	126.41	Upregulation
BF9343_0081	4-deoxy-L-threo-5-hexosulose-uronate ketol-isomerase	201.71	126.18	Upregulation

*^∗^After subtracting the transcript abundances in the wMtz from those determined for the Mtz8, the increased abundance in the treatment with metronidazole was related to the abundance in the wMtz that was normalized to 100%. Values > 100% means upregulation in Mtz8 and values < 100% means downregulation in Mtz8.*

## Conclusion

We have presented here a report providing a whole gene expression set related to response of *B. fragilis* ATCC 43859 exposed to SIC of metronidazole. In this approach, we selected from a wild type *B. fragilis* ATCC 43859 four derivative strains subcultured at 48 h intervals in the presence and absence of drug, under anaerobic conditions.

Overall, according to global gene expression by RNA-seq analysis, we found that the strains selected after metronidazole removal were functionally more similar to those selected after drug exposure, suggesting that even when the growth conditions are restored to the initial state without the drug, the recovered strain had drastic changes in their whole gene expression patterns.

**FIGURE 5:**
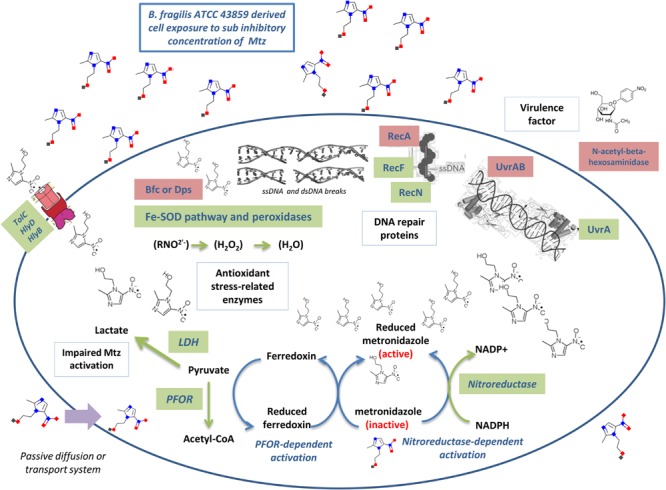
**Summarized *B. fragilis* response to metronidazole after exposure to SIC.** The mechanisms thought to be involved as described in this manuscript were ranging from metronidazole activation (via PFOR and nitroreductase), defense against superoxide ions (via antioxidant stress-related enzymes) or impaired reduction and activation of metronidazole (via LDH), high expression level of multidrug eﬄux pumps (TolC, HlyD, HlyB), and DNA repair (RecF, RecN, UvrA). Proteins names in green denote genes with high expression intensity whereas in red denote genes with low expression intensity under the exposure of metronidazole SIC.

This study addressed a broad range of issues related to the bacterial response to SIC of metronidazole. As shown in **Figure 5**, these mechanisms were ranging from metronidazole activation inside the cell (via PFOR and nitroreductase), defense against superoxide ions (via antioxidant stress-related enzymes) or impaired reduction and activation of metronidazole (via LDH), high expression level of multidrug eﬄux pumps (TolC, HlyD, HlyB), to DNA repair (RecF, RecN, UvrA). Mainly, we highlight that under the pressure of SIC of metronidazole, there seems to be an increased protection against nitro radical-anions mediated by peroxidases and Fe-SOD rather than for efficiency of DNA repair systems.

The results may help to elucidate the global response of *B. fragilis* to selective pressure imposed by metronidazole, especially at SICs, contributing with information about bacterial survival strategies under stress conditions in their environment. Despite the methodological limitations and high costs, this experimental design may be considered an important step, although a proteomic validation would be of interest, once some regulatory proteins are post-transcriptional regulated in prokaryotes, suggesting the need of caution in predicting protein coding sequences from only RNA-seq data. Further prospective studies are still needed to better address this issue, especially considering the genome coverage, and *in vivo* conditions, which would reflect an actual infection and antimicrobial chemotherapy.

## Author Contributions

MF, JR, and AF-M have contributed to the acquisition, analysis, interpretation of the data, and writing of the manuscript; GS has contributed to the analysis, interpretation of the data, and writing of the manuscript; AV has contributed to the analysis, and interpretation of the data; VS has contributed to the conception and design of the study, and writing of the manuscript; MN and CD have contributed to the conception and design of the study, analysis, interpretation of the data, and writing of the manuscript. All authors read and approved the final version of the manuscript.

## Conflict of Interest Statement

The authors declare that the research was conducted in the absence of any commercial or financial relationships that could be construed as a potential conflict of interest.

## Footnotes

1 http://geneontology.org

2 http://www.ncbi.nlm.nih.gov/COG

3 http://www.uniprot.org/help/uniprotkb

4 http://www.r-project.org

## Supplementary Material

FIGURE S1 **cDNA RNA-seq expression intensity of genes encoding for a PFOR, subunits and LDH in *B. fragilis* ATCC43859, throughout 48 h anaerobic subcultures**.

FIGURE S2 **cDNA RNA-seq expression intensity of genes *rec*A, *rec*F, *rec*N, and *ara*C-type in *B. fragilis* ATCC43859, throughout 48 h anaerobic subcultures**.

FIGURE S3 **cDNA RNA-seq expression intensity of genes *uvr*A1, *uvr*A2, *uvr*B, and *ara*C-type in *B. fragilis* ATCC43859, throughout 48 h anaerobic subcultures**.

TABLE S4 **Gene Ontology term (GO), biological process, molecular function or cellular location term of expressed genes identified for wMtz, Mtz2, Mtz8, rMtz2, rMtz8 strains**.

TABLE S5 **Shared core obtained among all tested *B. fragilis* strains selected in this study**.

TABLE S6 **K-means clustering and gene expression analyses to evaluate distinct gene expression patterns. Comparisons between different treatments were performed by edgeR.** Criteria for significance: *P*-value ≤ 0.05. Log fold-change (LogFC) values positives or negatives denote up or down-regulation, respectively in the second variable in comparisons.
