# Supplementary figures and images for: Exploratory Investigation of Bacteroides fragilis Transcriptional Response during In vitro Exposure to Subinhibitory Concentration of Metronidazole
Source: Front Microbiol. 2016 Sep 20;7:1465. doi: 10.3389/fmicb.2016.01465 (PMC5028390; doi:10.3389/fmicb.2016.01465)

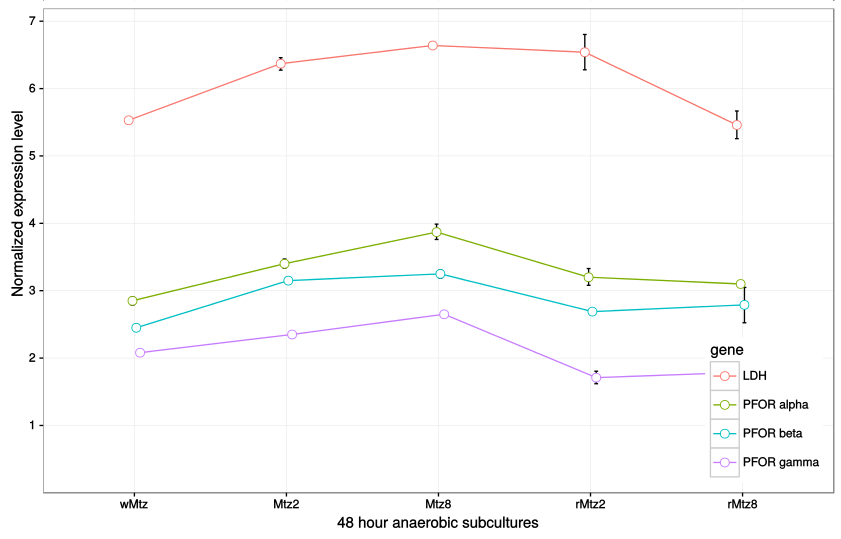

Supplement: FIGURE S1 — cDNA RNA-seq expression intensity of genes encoding for a PFOR, subunits and LDH in B. fragilis ATCC43859, throughout 48 h anaerobic subcultures. [file Image_1.TIF]

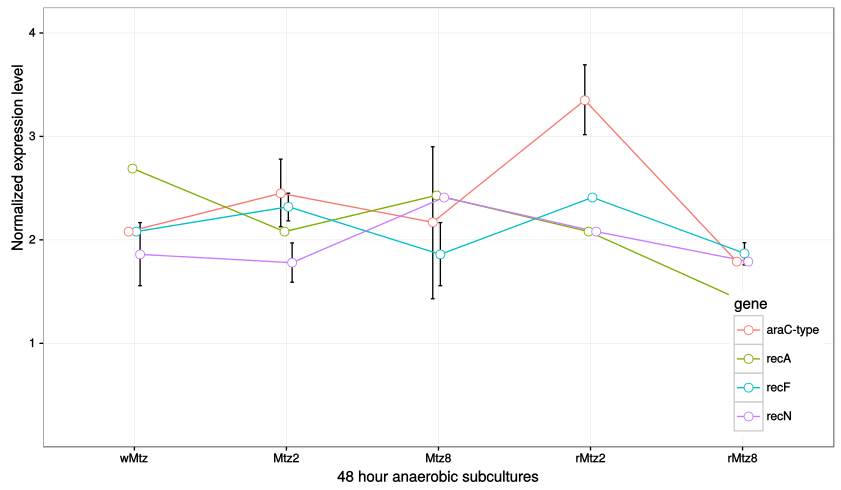

Supplement: FIGURE S2 — cDNA RNA-seq expression intensity of genes recA, recF, recN, and araC-type in B. fragilis ATCC43859, throughout 48 h anaerobic subcultures. [file Image_2.TIF]

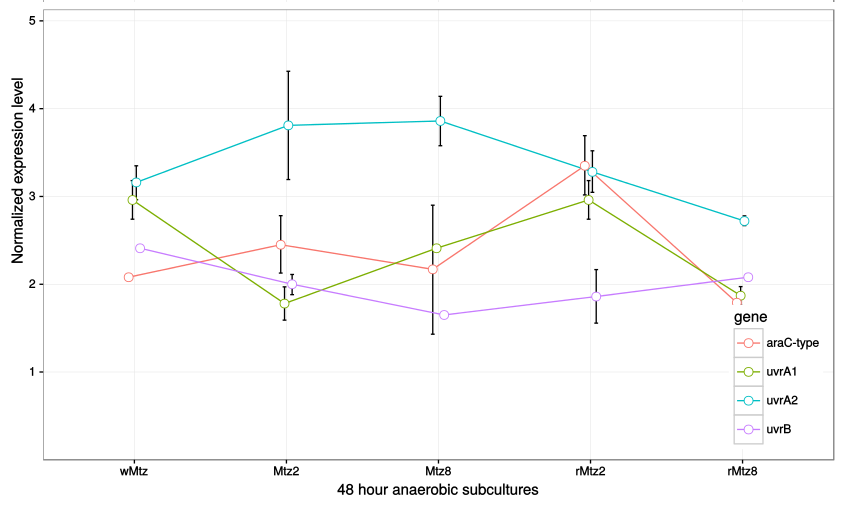

Supplement: FIGURE S3 — cDNA RNA-seq expression intensity of genes uvrA1, uvrA2, uvrB, and araC-type in B. fragilis ATCC43859, throughout 48 h anaerobic subcultures. [file Image_3.TIF]
